# Circular RNA TTC3 regulates cerebral ischemia-reperfusion injury and neural stem cells by miR-372-3p/TLR4 axis in cerebral infarction

**DOI:** 10.1186/s13287-021-02187-y

**Published:** 2021-02-12

**Authors:** Bo Yang, Li’e Zang, Jingwen Cui, Linlin Wei

**Affiliations:** 1 Department of Neurology, The First Affiliated Hospital of Jinzhou Medical University, Jinzhou, Liaoning Province China; 2 Department of Neurosurgery, The First Affiliated Hospital of Jinzhou Medical University, Jinzhou, Liaoning Province China; 3 Department of Gynaecology, The First Affiliated Hospital of Jinzhou Medical University, No.2, Section 5, Renmin Street, Jinzhou, Liaoning Province China

**Keywords:** Stroke, Cerebral ischemia/reperfusion injury, NSCs, circTTC3, miR-372-3p, TLR4

## Abstract

**Background:**

Stroke serves as a prevalent cerebrovascular disorder with severe cerebral ischemia/reperfusion (CIR) injury, in which neural stem cells (NSCs) play critical roles in the recovery of cerebral function. Circular RNAs (circRNAs) have been widely found to participate in stroke and NSC modulation. However, the role of circRNA TTC3 (circTTC3) in the regulation of CIR injury and NSCs remains elusive. Here, we aimed to explore the impact of circTTC3 on CIR injury and NSCs.

**Methods:**

The middle cerebral artery occlusion/repression (MCAO/R) model was established in C57BL/6J mice. The primary astrocytes were isolated from the cerebellum from C57BL/6J mice. The primary NSCs were obtained from rat embryos. The effect of circTTC3 on CIR injury and NSCs was analyzed by TTC staining, qPCR, Western blot, LDH colorimetric kits, MTT assays, Annexin V-FITC Apoptosis Detection Kit, luciferase reporter gene assays, and others in the system.

**Results:**

Significantly, the expression of circTTC3 was elevated in the MCAO/R mice and oxygen and glucose deprivation (OGD)-treated astrocytes. The depletion of circTTC3 attenuated cerebral infarction, neurological score, and brain water content. The OGD treatment induced apoptosis and the levels of lactate dehydrogenase (LDH) in the astrocytes, in which circTTC3 depletion reduced this phenotype in the system. Moreover, the depletion of circTTC3 promoted the proliferation and upregulated the nestin and β-tubulin III expression in NSCs. Mechanically, circTTC3 was able to sponge miR-372-3p, and miR-372-3p can target Toll-like receptor 4 (TLR4) in NSCs. The miR-372-3p inhibitor or TLR4 overexpression could reverse circTTC3 depletion-mediated astrocyte OGD injury and NSC regulation.

**Conclusion:**

Thus, we conclude that circTTC3 regulates CIR injury and NSCs by the miR-372-3p/TLR4 axis in cerebral infarction. Our finding presents new insight into the mechanism by which circTTC3 modulates CIR injury and NSC dysfunction. CircTTC3, miR-372-3p, and TLR4 may serve as potential targets for the treatment of CIR injury during stroke.

## Background

Stroke serves as a leading cause of severe mortality and disability globally [1, 2]. Nearly 80 to 85% of strokes are caused by cerebral ischemia, commonly induced by thromboembolism and embolism occlusion of the primary brain aorta [1–3]. Cerebral ischemia-reperfusion (CIR) injury is the neurotic disorder with neuro-destruction induced by hypoxia and ischemia, which is additionally intensified by the short-term blood perfusion recovery [4, 5]. An increasing collection of evidence indicates that CIR injury usually comprises a variety of damaging processes, including inflammation and oxidative stress, eventually resulting in acute autophagy, apoptosis, and necrosis in the ischemic cerebrum [6–8]. Therefore, it is urgent to understand the molecular mechanism of CIR injury. Moreover, neural stem cells (NSCs) described as precursor cells with self-renew capacity are able to differentiate to neural cells, such as neurons and astrocytes [9, 10]. It has been identified that NSCs benefit adjuncts and are potentially applied in the treatment of cerebral infarction [11, 12]. However, the understanding of NSC regulation during CIR injury remains limited.

Given the advanced development of new-generation sequencing technology, circular RNAs (circRNAs) have been identified as a critical kind of regulatory RNAs that form a loop structure without 5′-3′ polyadenylated or polarities tails [13, 14]. Meanwhile, circRNA TTC3 (circTTC3) has been reported to participate in the regulation of hypoxic injury [15]. MicroRNAs (miRNAs) serve as a 17–25-nucleotide small non-coding RNA and demonstrate critical functions in various biological processes by targeting related genes [16–18]. In addition, circRNAs and miRNAs play critical functions in the modulation of CIR injury and NSCs [19–22]. However, the effect of circTTC3 and miR-372-3p on CIR injury and NSCs is still unclear. Furthermore, TLR4 has been identified to be involved in regulating NSCs during stroke progression [23], but the correlation of TLR4 with circTTC3 and miR-372-3p remains elusive.

In this study, we were interested in the function of circTTC3 in CIR injury and NSC modulation. We identified a novel role of circTTC3 in regulating CIR injury and NSCs by the miR-372-3p/TLR4 axis in cerebral infarction.

## Methods

### Middle cerebral artery occlusion/reperfusion mouse model

To analyzed CIR injury, the middle cerebral artery occlusion/repression (MCAO/R) model was established in C57BL/6J mice (20–25 g, male, 6 weeks old) as previously reported [24]. Briefly, the mice (*n* = 5) were anesthetized by 30 mg/kg sodium pentobarbital, fixed on an operating heating table, and incubated in a ventilator to sustain life. The mice were incised to expose the right-side common carotid artery, and a 4-0 nylon filament was inserted into the end of the internal carotid artery from the common carotid artery through the external carotid artery to occlude the blood. The blood occlusion was administrated for 2 h, then the blood supplement was restored for 24 h. The mice of the sham group received a similar operation with no MCAO/R occlusion. The mice were intracerebroventricularly injected with control shRNA or circTTC3 shRNA (before MCAO/R occlusion) as in the previous reports [25, 26]. Then, the mice succumbed to death, and the brain tissues were obtained for further analysis. The neurological function score and brain water content were measured after reperfusion. Animal care and method procedure were authorized by the Animal Ethics Committee of The First Affiliated Hospital of Jinzhou Medical University.

### TTC staining

The cerebral infarction was assessed by employing 2,3,5-triphenyltetrazolium chloride (TTC, Sigma, USA) staining. Shortly, the brain tissues were cut into 2-mm slices and cultured by 2% TTC solution for 20 min. Then, the samples were fixed by 4% paraformaldehyde. The control tissues were stained by red with TTC, while the cerebral infarction was unstained. The cerebral infarction was calculated and quantified.

### Cell culture and treatment

The primary astrocytes were isolated from the cerebellum from C57BL/6J mice and cultured in DMEM medium (Gibco, USA) with 10% FBS (Gibco, USA) at the condition of 5% CO_2_ and 37 °C as described previously [26]. The levels of TNF-α and IL-1β were analyzed by ELISA kits (Sangon Biotech, China). Primary NSCs were obtained from rat embryos and cultured in DMEM medium with basic fibroblast growth factor, epidermal growth factor (EGF) (Sigma, USA), and N2 as previously reported [27]. The NSCs were identified by neurosphere formation and the expression of Nestin and Sox2 as in the previous reports [28–32]. The control shRNA, circTTC3 shRNA, pcDNA-TLR4, miR-372-3p mimic, and miR-372-3p inhibitor were transfected in the cells using Lipofectamine 3000 (Invitrogen, USA), according to the manufacturer’s instructions.

Oxygen glucose deprivation-reperfusion (OGD/R) was carried out as previously reported [25]. In brief, cells were cultured in DMEM without FBS and glucose in an incubator for 3 h with premixed gas (5% CO_2_ and 95% N_2_). Then, the cells were given normal DMEM with 10% FBS and placed in a CO_2_ incubator (5% CO_2_ and 95% air). Cells in the control group were cultured with normal DMEM and 10% FBS for the same incubation times.

### Quantitative reverse transcription-PCR

The isolation of RNAs was performed by applying TRIzol Reagent (Solarbio, China) and the first-strand cDNA was synthesized (Solarbio, China). The qRT-PCR was carried out by applying SYBR-Green (Takara, China). The standard control for mRNA and miRNA was GAPDH and U6, respectively. Relative expression was calculated using the 2^−ΔΔCt^ method. The primer sequences are as follows: circTTC3 forward 5′-CCTGTGTAGAAGCCATCCGT-3′, reverse 5′-ATCATCAGTGGTAAAGTCAGGAGTA-3′; miR-372-3p, forward 5′-TTTCACGACGCTGTAAACTCGCA-3′; TLR4, forward 5′-TTGTTTCGCAAGCTTCCGTT-3′, reverse 5′-ACGTGGGCATTTGTCACGAT-3′; Nestin, forward 5′-GATCTAAACAGGAAGGAAATCCAGG-3′, reverse 5′-TCTAGTGTCTCATGGCTCTGGTTTT-3′; Sox2, forward 5′-CACAACTCGGAGATCAGCAA-3′, reverse 5′-CGGGGCCGGTATTTATAATC-3′; β-tubulin III, forward 5′-AGCAAGGTGCGTGAGGAGTA-3′, reverse 5′-TCTAGTGTCTCATGGCTCTGGTTTT-3′; GAPDH, forward 5′-AACGGATTTGGTCGTATTGGG-3′, reverse 5′-CCTGGAAGATGGTGATGGGAT-3′; and U6, forward 5′-GCTTCGGCAGCACATATACTAA-3′, reverse 5′-AACGCTTCACGAATTTGCGT-3′.

### Western blot analysis

Total proteins were extracted from the cells using RIPA buffer (CST, USA) and quantified using the BCA Protein Quantification Kit (Abbkine, USA). The proteins at the same concentration were subjected to SDS-PAGE and transferred in PVDF membranes (Millipore, USA), followed by the incubation with 5% milk and with the primary antibodies at 4 °C overnight. The corresponding second antibodies (BOSTER, China) were used for incubating the membranes for 1 h at room temperature, followed by the visualization by using a chemiluminescence detection kit (Beyyotime, China). The primary antibodies applied in this study comprise TLR4 (Abcam, USA), β-tubulin III (Abcam, USA), Bax (Abcam, USA), Bcl-2 (Abcam, USA), Caspase-3 (Abcam, USA), H2AX (Abcam, USA), γH2AX (Abcam, USA), and β-actin (Abcam, USA).

### Senescence analysis

The senescence was analyzed by the quantitative senescence-associated β-galactosidase assays as in the previous report [33]. Briefly, 4-methylumbelliferyl-β-d-galactopyranoside (4-MUG) was a β-galactosidase substrate that did not emit fluorescence until cleaved by the enzyme to generate the fluorophore 4-methylumbelliferone. As already reported, we performed an assay on cell lysates to monitor the fluorophore production at an emission/excitation wavelength of 365/460 nm.

### LDH activity

The lactate dehydrogenase (LDH) activities were analyzed by utilizing LDH colorimetric kits (Jiancheng Biotechnology, China) according to the manufacturers’ guidelines in the culture medium of the cells.

### MTT assay

The cell viability was assessed by MTT assays at the indicated times in 6-well dishes. Briefly, the MTT solution (Solarbio, China) was plused in the cells and incubated for 4 h at 5% CO_2_, 37 °C. Afterward, dimethyl sulfoxide (DMSO, 100 μL, 10 min, Sigma, USA) was applied to terminate the reaction. The cell viability was measured at the absorbance of 490 nm by applying the microplate reader (Thermo, USA).

### Analysis of cell apoptosis

About 2 × 10^5^ cells were plated on 6-well dishes. Cell apoptosis was analyzed using the Annexin V-FITC Apoptosis Detection Kit (KeyGen, China) following the manufacturer’s instructions. Briefly, about 2 × 10^5^ cells were collected by binding buffer and dyed at 25 °C, followed by the flow cytometry analysis.

### Bioinformatic analysis

The potential interaction between circTTC3 and miR-372-3p was identified by the bioinformatic analysis using ENCORI (http://starbase.sysu.edu.cn/index.php). The interaction of miR-372-3p and TLR4 3′ UTR was identified by bioinformatic analysis using Targetscan (http://www.targetscan.org/vert_72/).

### Luciferase reporter gene assay

The luciferase reporter gene assays were carried out by using the Dual-luciferase Reporter Assay System (Promega, USA). The cells were transfected with the pmirGLO-TLR4 or pmirGLO-circTTC3, and L miR-372-3p mimic by applying riboFECT™ CP Transfection Kit (RiboBio, China), followed by the analysis of luciferase activities based on the Dual-luciferase Reporter Assay System (Promega, USA). As a control, the luciferase activities of Renilla were measured.

### Statistical analysis

Data were presented as mean ± SD, and the statistical analysis was performed by GraphPad prism 7. The unpaired Student’s *t* test was applied for comparing two groups, and one-way ANOVA was applied for comparing among multiple groups. *P* < 0.05 were considered as statistically significant.

## Results

### The expression of circTTC3 is elevated in the MCAO/R mice and enhances cerebral infarction in vivo

**Fig. 1:**
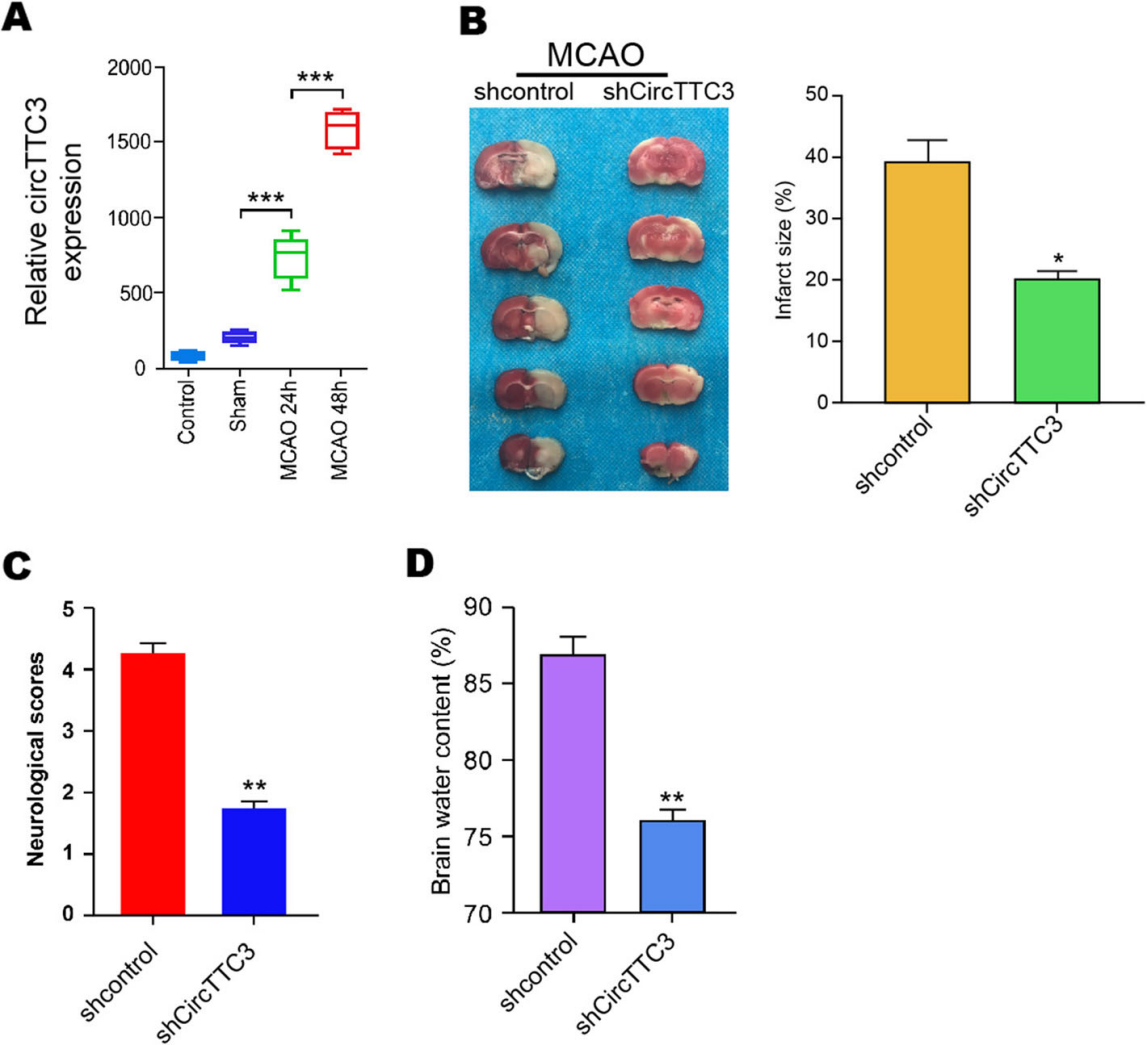
The expression of circTTC3 is elevated in the MCAO/R mice and enhances cerebral infarction in vivo. **a** The MCAO/R mouse model was constructed, and the mice were intracerebroventricularly injected with control shRNA or circTTC3 shRNA. **a** The expression of circTTC3 was measured by qPCR in the mice. **b** The cerebral infarction was analyzed by TTC staining in the mice. **c**, **d** The neurological deficit scores and brain water content were measured in the mice. Data are presented as mean ± SD. Statistic significant differences were indicated: *P < 0.05, **P < 0.01

To understand the potential correlation of circTTC3 with CIR injury, we established a middle cerebral artery occlusion/repression (MCAO/R) mouse model, and the mice were intracerebroventricularly injected with control shRNA or circTTC3 shRNA. The expression of circTTC3 was significantly elevated in the MCAO/R mice compared with that in the control mice (Fig. 1a). TTC staining revealed that the depletion of circTTC3 repressed cerebral infarction in the MCAO/R mice (Fig. 1b). Meanwhile, the neurological score and brain water content were reduced by circTTC3 knockdown in the mice (Fig. 1c, d). Together, these suggest that circTTC3 enhances cerebral infarction in vivo.

### CircTTC3 promotes OGD-induced astrocyte injury in vitro

**Fig. 2:**
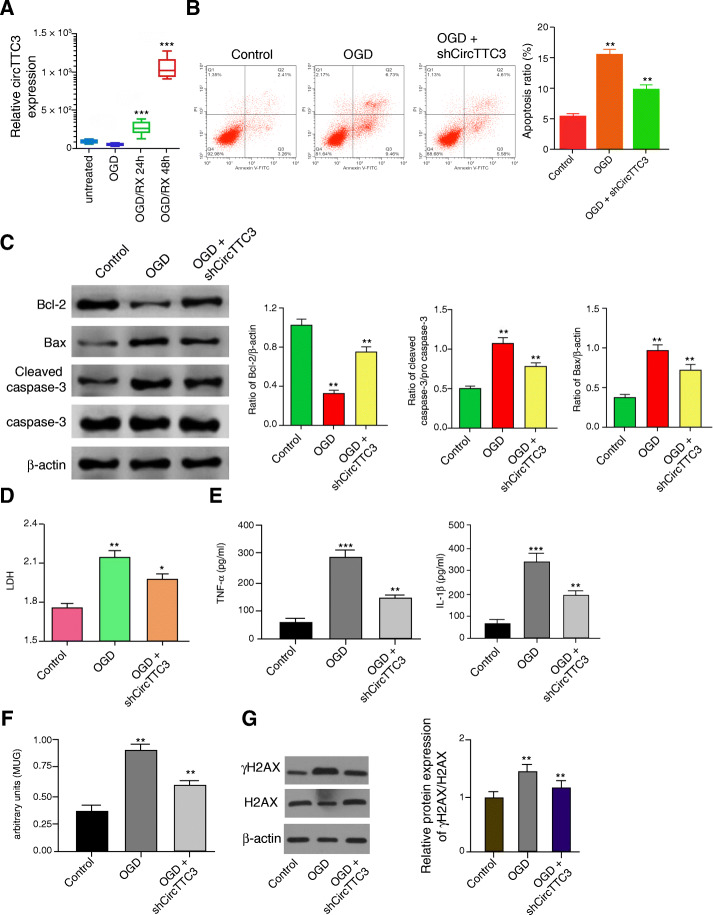
CircTTC3 promotes OGD-induced astrocyte injury in vitro. **a** The astrocytes were treated with OGD and the expression of circTTC3 was measured by qPCR in the cells. **b**–**g** The OGD-treated astrocytes were treated with control shRNA or circTTC3 shRNA. **b** The cell apoptosis was assessed by flow cytometry analysis in the cells. **c** The expression levels of Bax, Bcl-2, caspase-3, cleaved caspase-3, and β-actin were determined by Western blot analysis in the cells. The results of Western blot analysis were quantified by the ImageJ software. **d** The levels of LDH in the culture medium were assessed by colorimetric assays in the cells. **e** The levels of TNF-α and IL-1β were analyzed by ELISA assays. **f** Senescence was evaluated by MUG assays. **g** The protein expression of H2AX, γH2AX, and β-actin was determined by Western blot analysis in the cells. The results of Western blot analysis were quantified by the ImageJ software. Data are presented as mean ± SD. Statistic significant differences were indicated: *P < 0.05, **P < 0.01

**Fig. 3:**
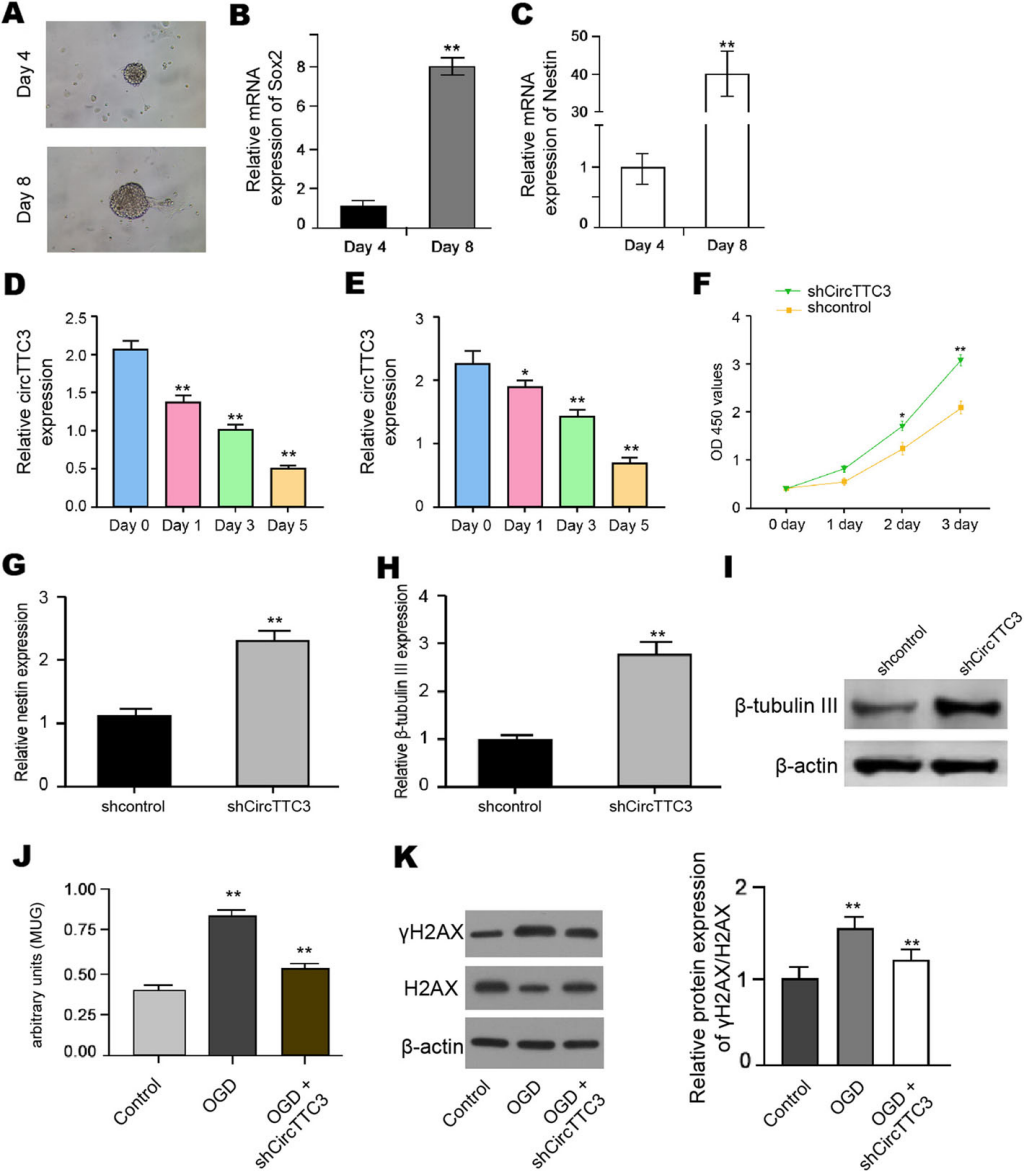
CircTTC3 inhibits proliferation and differentiation of NSCs. **a** The NSCs were identified by neurosphere formation. **b**, **c** The expression of Nestin and Sox2 was measured by qPCR in NSCs. **d**, **e** The expression of circTTC3 was measured by qPCR during NSC differentiation into astrocytes and neurons, respectively. **f**–**i** The NSCs were treated with control shRNA or circTTC3 shRNA. **f** The cell viability was measured by MTT assays in the cells. **g** The expression of nestin was analyzed by qPCR in the cells. **h, i** The mRNA and protein β-tubulin III expression were tested by qPCR and Western blot analysis, respectively. **j** Senescence was evaluated by MUG assays. **k** The protein expression of H2AX, γH2AX, and β-actin was determined by Western blot analysis in the cells. The results of Western blot analysis were quantified by the ImageJ software. Data are presented as mean ± SD. Statistic significant differences were indicated: *P < 0.05, **P < 0.01

Next, we analyzed the expression of circTTC3 in the OGD-treated astrocytes. Significantly, we found that circTTC3 expression was upregulated in the OGD-treated astrocytes (Fig. 2a). Moreover, the OGD treatment induced apoptosis in the astrocytes, in which the depletion of circTTC3 reduced this phenotype in the system (Fig. 2b). Similarly, the Bcl-2 expression was inhibited, and Bax and cleaved caspase-3 expression were increased in the OGD-treated astrocytes, while circTTC3 knockdown could reverse this effect (Fig. 2c). In addition, the levels of lactate dehydrogenase (LDH) in the culture medium were remarkably enhanced by the OGD treatment, which were decreased by circTTC3 depletion in the cells (Fig. 2d). Meanwhile, the OGD treatment enhanced the levels TNF-α and IL-1β in the culture medium, in which the circTTC3 depletion attenuated the phenotype (Fig. 2e). Moreover, MUG assays revealed that the senescence was enhanced by OGD treatment, in which circTTC3 depletion could reverse this effect (Fig. 3f). Besides, the expression levels of γH2AX were decreased by the depletion of circTTC3 in the OGD-treated NSCs (Fig. 3g). These data indicate that circTTC3 promotes OGD-induced astrocyte injury in vitro*.*

### CircTTC3 inhibits proliferation and differentiation of NSCs

Then, we further evaluated the effect of circTTC3 on NSC regulation. The NSCs were identified by neurosphere formation and the expression of Nestin and Sox2 (Fig. 3a–c). Our data showed that the expression of circTTC3 was decreased during NSC differentiation into both astrocytes and neurons (Fig. 3d, e). Significantly, the depletion of circTTC3 increased the proliferation of NSCs (Fig. 3f). The expression of nestin was upregulated by circTTC3 knockdown in the cells (Fig. 3g). Moreover, the circTTC3 depletion remarkably enhanced β-tubulin III expression (Fig. 3h, i). Interestingly, MUG assays showed that the senescence was induced by OGD treatment while circTTC3 depletion was able to attenuate this phenotype (Fig. 3j). In addition, the expression levels of γH2AX were reduced by the depletion of circTTC3 in the OGD-treated NSCs (Fig. 3k). Taken together, circTTC3 is able to repress the proliferation and differentiation of NSCs.

### CircTTC3 is able to sponge miR-372-3p in NSCs

**Fig. 4:**
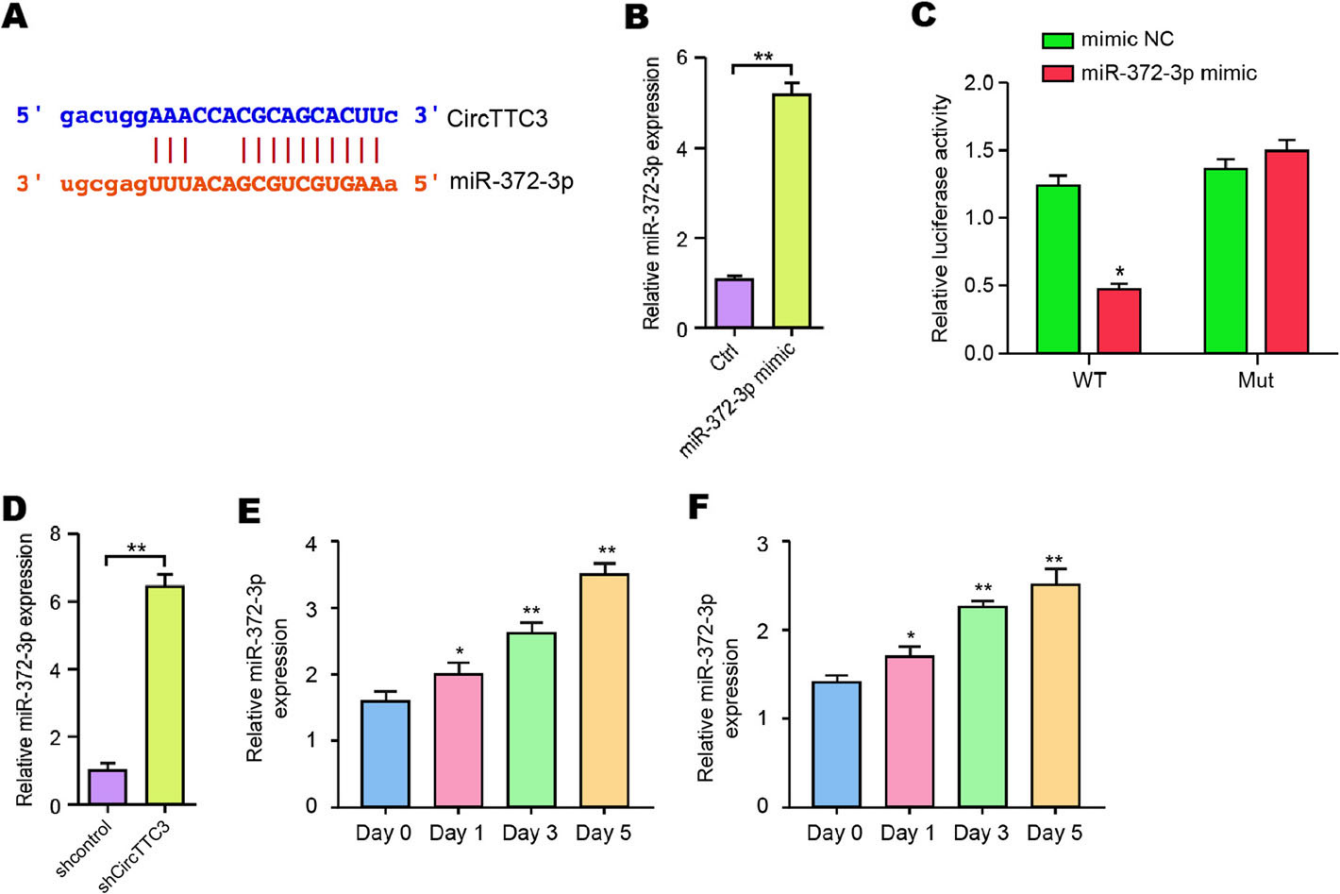
CircTTC3 is able to sponge miR-372-3p in NSCs. **a** The potential interaction between circTTC3 and miR-372-3p was identified by the bioinformatic analysis using ENCORI (http://starbase.sysu.edu.cn/index.php). **b**, **c** The NSCs were treated with the miR-372-3p mimic or control mimic. **b** The expression levels of miR-372-3p were measured by qPCR in the cells. **c** The luciferase activities of wild-type circTTC3 (WT) and circTTC3 with the miR-372-3p-binding site mutant (MUT) were determined by luciferase reporter gene assays in the cells. **d** The NSC cells were infected with lentiviral plasmids carrying circTTC3 shRNA or corresponding control shRNA. The expression of miR-372-3p was analyzed by qPCR in the cells. **e**, **f** The expression of miR-372-3p was measured by qPCR during NSC differentiation into astrocytes and neurons, respectively. Data are presented as mean ± SD. Statistic significant differences were indicated: *P < 0.05, **P < 0.01

Next, we identified the potential interaction between circTTC3 and miR-372-3p in the bioinformatic analysis (Fig. 4a). Then, we treated the NSCs with miR-372-3p mimic, and the efficiency was confirmed in the cells (Fig. 4b). Significantly, miR-372-3p mimic decreased the luciferase activities of circTTC3 but not the circTTC3 mutant (Fig. 4c). Moreover, NSCs were infected with lentiviral plasmids carrying circTTC3 shRNA or corresponding control shRNA. Notably, the depletion of circTTC3 enhanced the expression of miR-372-3p in the cells (Fig. 4d). Moreover, we validated that miR-372-3p was increased during NSC differentiation into both astrocytes and neurons (Fig. 4e, f). These data indicate that circTTC3 is able to sponge miR-372-3p in NSCs.

### miR-372-3p is able to target TLR4 in NSCs

**Fig. 5:**
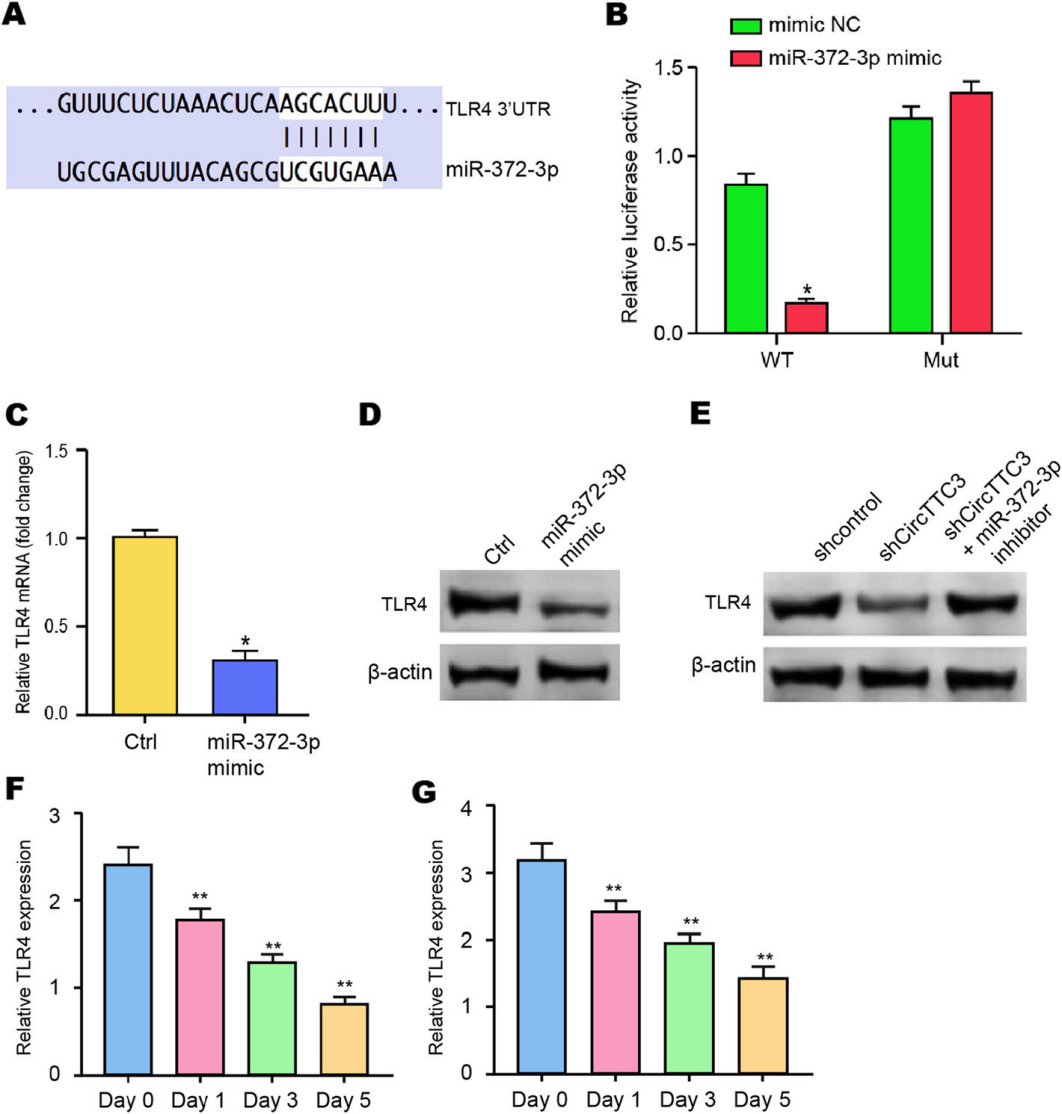
miR-372-3p is able to target TLR4 in NSCs. **a** The interaction of miR-372-3p and TLR4 3′ UTR was identified by bioinformatic analysis using Targetscan (http://www.targetscan.org/vert_72/). **b**–**d** The NSCs were treated with the miR-372-3p mimic or control mimic. **b** The luciferase activities of wild-type TLR4 (WT) and TLR4 with the miR-372-3p-binding site mutant (MUT) were determined by luciferase reporter gene assays in the cell. **c** The mRNA expression of TLR4 was analyzed by qPCR in the cells. **d** The protein expression of TLR4 and β-actin was tested by Western blot analysis in the cells. **e**–**g** The NSCs were treated with control shRNA or circTTC3 shRNA or co-treated with circTTC3 shRNA and miR-372-3p inhibitor. The protein expression of TLR4 and β-actin was tested by Western blot analysis in the cells. Data are presented as mean ± SD. Statistic significant differences were indicated: *P < 0.05, **P < 0.01

We then explored the target of miR-372-3p in the NSCs. We identified the miR-372-3p-targeted site in TLR4 3′ UTR based on bioinformatic analysis (Fig. 5a). To determine the impact of miR-372-3p on TLR4, we treated the NSC cells with miR-372-3p mimic and found that the miR-372-3p mimic treatment inhibited luciferase activities of wild-type TLR4 but not the TLR4 mutant in the NP cells (Fig. 5b). Moreover, the mRNA and protein expression of TLR4 were significantly reduced by miR-372-3p mimic in NSCs (Fig. 5c, d), suggesting that miR-372-3p is able to target TLR4 in the NSCs. Meanwhile, the depletion of circTTC3 downregulated the TLR4 expression, in which miR-372-3p inhibitor could reverse this effect in the system (Fig. 5e). Besides, the expression of TLR4 was repressed during NSC differentiation into both astrocytes and neurons (Fig. 5f, g). Taken together, our results suggest that miR-372-3p is able to target TLR4 in NSCs.

### CircTTC3 regulates OGD-induced astrocyte injury and NSCs by miR-372-3p/TLR4 axis

**Fig. 6:**
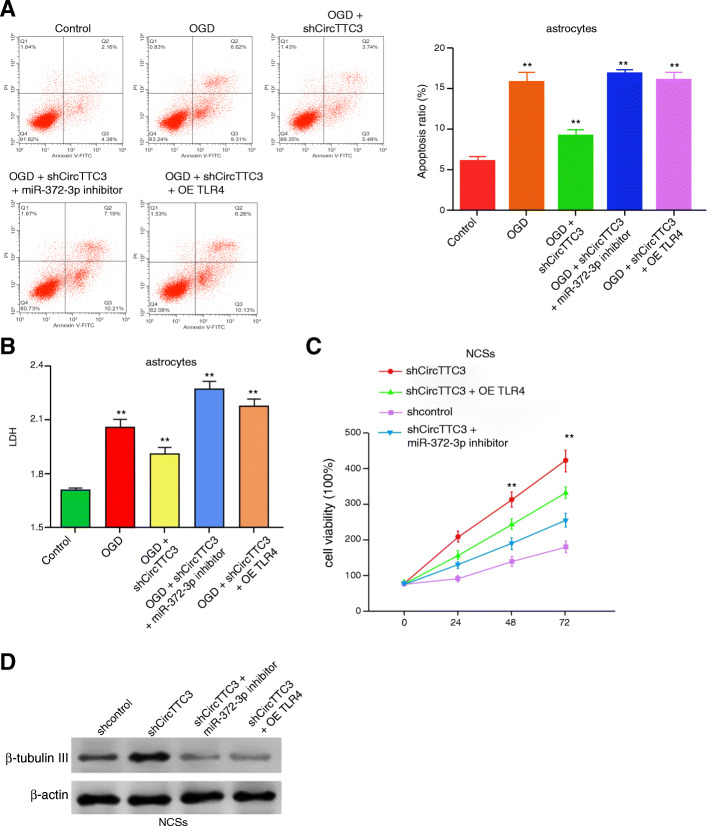
CircTTC3 regulates OGD-induced astrocyte injury and NSCs by the miR-372-3p/TLR4 axis. **a**, **b** The OGD-treated astrocytes were treated with control shRNA or circTTC3 shRNA or co-treated with circTTC3 shRNA and miR-372-3p inhibitor or pcDNA-TLR4. **a** The cell apoptosis was assessed by flow cytometry analysis in the cells. **b** The levels of LDH in the culture medium were assessed by colorimetric assays in the cells. **c**, **d** The NSCs were treated with control shRNA or circTTC3 shRNA or co-treated with circTTC3 shRNA and miR-372-3p inhibitor or pcDNA-TLR4. **c** The cell viability was measured by MTT assays in the cells. **d** The protein β-tubulin III expression were tested by qPCR and Western blot analysis, respectively. Data are presented as mean ± SD. Statistic significant differences were indicated: **P < 0.01

Next, we tried to explore the role of the circTTC3/miR-372-3p/TLR4 axis in the CIR injury and NSC modulation. Our data demonstrated that the depletion of circTTC3 was able to inhibit apoptosis and LDH levels in the astrocytes, in which the miR-372-3p inhibitor or TLR4 overexpression could reverse this effect in the system (Fig. 6a, b). Meanwhile, circTTC3 knockdown enhanced the viability of NSCs, while the miR-372-3p inhibitor or TLR4 overexpression was able to block this phenotype in the NSCs (Fig. 6c). Furthermore, the expression of β-tubulin III was upregulated by circTTC3 depletion, in which miR-372-3p inhibitor or TLR4 overexpression reduced the effect (Fig. 6d). These data indicate that circTTC3 regulates OGD-induced astrocyte injury and NSCs by the miR-372-3p/TLR4 axis.

## Discussion

Stroke is a common cerebrovascular disorder with severe CIR injury, in which NSCs play critical roles [34]. circRNAs have been found to present crucial functions in the regulation of CIR injury and NSCs [19, 20]. In this study, we found that circTTC3 was able to promote CIR injury and inhibit NSC proliferation and differentiation by the miR-372-3p/TLR4 axis in cerebral infarction.

Several previous studies have found that circRNAs play crucial functions in the modulation of CIR injury and NSCs. It has been reported that circ008018 inhibition attenuates CIR injury by repressing miR-99a [35]. Circcamk4 contributes to CIR-induced neuronal injury [36]. CircUCK2 elevation attenuates apoptosis by miR-125b-5p/GDF11 axis in CIR injury [37]. The suppression of circ HIPK2 regulates function recovery after ischaemic stroke in NSCs [38]. Moreover, it has been shown that circTTC3 modulates heart function by targeting miR-15b in myocardial infarction [39]. CircTTC3 inhibits hypoxic damage of HaCaT cells by sponging miR-449a [15]. In the present study, we first identified that the expression of circTTC3 was upregulated in MCAO/R mice and OGD-treated astrocytes. circTTC3 was able to enhance cerebral infarction in vivo and promote OGD-induced astrocyte injury in vitro*.* Moreover, circTTC3 was downregulated during the differentiation of NSCs and inhibited proliferation and the differentiation of NSCs. Our data demonstrated an important role of circTTC3 in regulating CIR injury and NSCs, presenting informative evidence for the essential function of circRNAs in stroke.

Furthermore, multiple miRNAs are reported to participate in the mediation of CIR injury and NSCs. MiRNA-182-5p inhibits CIR injury by repressing the Toll-like receptor 4 [40]. The elevation of miR-496 suppresses CIR injury by negatively modulating BCL2L14 [41]. MiRNA-424 alleviates CIR injury by inhibiting oxidative stress in mice [42]. MiR-7 modulates nerve damage repair by regulating NSC proliferation and differentiation by targeting cdc42 [43]. MiRNA-145 modulates the differentiation of NSCs by the Sox2-Lin28/let-7 axis [44]. Moreover, it has been reported that the upregulation of TLR4 is able to attenuate the CIR injury and serves as a negative modulator of NSCs [45, 46]. In the mechanism research of this study, circTTC3 was able to sponge miR-372-3p and miR-372-3p could target TLR4 in NSCs. The miR-372-3p inhibitor or TLR4 overexpression could reverse circTTC3 depletion-mediated astrocyte OGD injury and NSC regulation. These results indicate an unreported correlation of circTTC3 with miR-372-3p and TLR4, implying a new mechanism involving circTTC3, miR-372-3p, and TLR4 during stroke.

In conclusion, we identified that circTTC3 regulated CIR injury and NSCs by the miR-372-3p/TLR4 axis in cerebral infarction. Our finding presents new insight into the mechanism of circTTC3 modulates CIR injury and NSC dysfunction. CircTTC3, miR-372-3p, and TLR4 may serve as potential targets for the treatment of CIR injury during stroke.

## Data Availability

The datasets used and/or analyzed during the current study are available from the corresponding author on reasonable request.

## Abbreviations

CIR: Cerebral ischemia/reperfusion
NSCs: Neural stem cells
circRNAs: Circular RNAs
circTTC3: circRNA TTC3
MCAO/R: Middle cerebral artery occlusion/repression
OGD: Oxygen and glucose deprivation
LDH: Lactate dehydrogenase
EGF: Epidermal growth factor
TTC: Triphenyltetrazolium chloride
TLR4: Toll-like receptor 4
